# Current Status and Trends of the Cement Admixtures

**DOI:** 10.3390/ma19010187

**Published:** 2026-01-04

**Authors:** Hongwei Wang, Ying Shi

**Affiliations:** School of Resources and Safety Engineering, Central South University, Changsha 410083, China; hongwei.wang@csu.edu.cn

Cement-based materials are central to modern infrastructure construction. However, their production and application are accompanied by significant resource consumption and environmental emissions. To achieve sustainable development in the construction sector, optimizing cement performance, enhancing durability, and reducing environmental impact through admixture technology have become important research directions [1].

Current research on cement-based materials primarily follows two directions: investigating microscopic mechanisms and developing novel admixtures. At the micro-mechanism level, progress in experimental techniques, interdisciplinary approaches, and computational science has provided strong support for studying the hydration processes and gel properties of cement-based materials. For instance, Wang et al. [2] applied the Krstulović–Dabić kinetic model and GEMS thermodynamic analysis to show that a low-heat-release polymer retards cement hydration by “inhibiting nucleation and growth processes and delaying gypsum consumption.” Golewski [3] used scanning electron microscopy to demonstrate that quaternary blended cements containing silica fume, fly ash, and nanosilica can reduce the width of microcracks in the interfacial transition zone by up to 48%, mainly due to the “homogenization of the composite structure and limitation of initial internal damage in concrete.” Through X-ray spectral microanalysis, diffraction granulometric analysis, and rheological tests, Klyuev et al. [4] showed that low-cement mortars with fly ash, crushed quartzite sandstone, and PFM-NLK superplasticizer achieve a 70% reduction in cement content while reaching 28-day compressive and flexural strengths of 62.9 MPa and 7.9 MPa, respectively, exceeding conventional cement by 23% and 52%. These mortars also exhibit “suitability for 3D printing applications” owing to their optimized particle size distribution and flowability. The continuous output of research findings is further deepening this field. For example, based on a comprehensive literature review, Dahlan [5] concluded that nanotechnology, particularly the incorporation of nanoparticles such as SiO_2_, TiO_2_, and carbon nanotubes, “significantly enhances the performance of cement and concrete” by improving mechanical strength and durability, as well as introducing multifunctional properties like self-cleaning and piezoresistivity. Among these, the fracture mode of materials directly determines their engineering and safety performance, constituting a key area of research at the microscopic level: Golewski [6] demonstrated through SEM analysis that incorporating 20% fly ash minimizes interfacial transition zone (ITZ) crack width to 300 nm in mature concrete, thereby enhancing durability and potentially reducing hazardous fly ash disposal by 160 million tons annually.

The in-depth exploration of microstructural characteristics has directly spurred numerous interdisciplinary collaborations, leading to the emergence of novel external additives that enhance the performance of cement-based materials: Dvorkin et al. [7] demonstrate, through experimental design, regression analysis, and high-temperature testing, that the mechano-chemical activation of cement–ash binders by increasing fly ash specific surface area and introducing a 1% Na_2_SiF_6_ additive significantly could enhances the properties of heat-resistant mortars. Yang et al. [8] studied the swelling behavior of a superabsorbent polymer in cementitious environments, finding that its absorptivity is critically influenced by factors including mix composition, superplasticizer dosage, stirring rate, and curing conditions. Whilst enhancing the performance of cementitious materials remains the core objective, their integration with solid waste and environmentally friendly materials can significantly amplify their environmental benefits. This approach has garnered widespread attention in the global drive towards industrial sustainability: Klyuev et al. [9] investigated the utilization of molten spherical basalt particles (beads), a waste product from mineral wool production, as an active mineral additive in cement paste, and found that finely ground beads, particularly at 5–10% replacement, exhibit pozzolanic activity by absorbing portlandite and contribute to a densified microstructure, thereby enhancing the composite’s compressive strength. Aiken et al. [10] found that partially replacing MgO in magnesium oxychloride cement with supplementary materials, especially metakaolin, significantly enhances its water resistance, primarily through physical particle packing effects rather than new chemical reactions. Zhao et al. [11] developed a low-carbon binder for cemented paste backfill using low-reactivity blast furnace slag, superfine fly ash, and chemical additives (aluminum sulfate and carbide slag), which significantly enhanced compressive strength through both physical filling and chemical activation. Chernysheva et al. [12] confirmed through XRD, SEM, and DTA analyses that incorporating mechanically activated thermal power plant waste (fly ash/slag) and microfibers (polyamide/basalt) into gypsum–cement binders results in a densified microstructure and enhanced mechanical properties. Bio-based additives, exemplified by biochar, confer novel green benefits to cement-based materials: Wei et al. [13] demonstrate that pyroligneous acid (PA), a bio-additive from applewood pyrolysis, effectively enhances strength and retards setting in both sulfoaluminate and Portland cements at low doses (≤2%), as determined through performance evaluation and microstructural characterization, demonstrating its potential as a sustainable alternative to conventional chemical admixtures. Chen et al. [14] demonstrated that incorporating carbon-negative biochars (rice husk and yard waste) as green additives in cementitious binders enhanced both hydration via internal curing/pozzolanic reactions and the immobilization efficiency of toxic elements in municipal solid waste incineration fly ash, as evidenced by multi-technique characterization (TGA, XRD, NMR, etc.), thereby advancing low-carbon stabilization/solidification technology for hazardous waste treatment. Concurrently, the rapid advancement of machine learning has opened new avenues for verifying the performance of cementitious materials: Han et al. [15] demonstrated through experimental tests and an Adaptive Neuro-Fuzzy Inference System (ANFIS) that volcanic powder effectively maintains slump flow while the combination of micro-silica and volcanic powder in ternary mixes optimally enhances compressive strength, electrical resistivity, and durability of self-consolidating green concrete with partial cement replacements.

Cement-based materials have reached a relatively advanced stage of development. Nevertheless, driven by interdisciplinary integration and emerging technological innovations, this field is once again demonstrating remarkable research vitality and innovation potential. This Special Issue, centered on the theme “Effects of Adding Cement Admixtures on the Microstructure and Properties of Cement Materials,” compiles several cutting-edge studies. It covers aspects such as mechanical property enhancement, micro-mechanism analysis, development of novel additives, and environmental co-benefits, aiming to provide the latest scientific and technological trends and theoretical references for scholars and engineers in the field.

Mechanical strength is a fundamental indicator for evaluating the performance of cement-based materials, directly impacting engineering safety and service life. Multiple articles in this issue focus on strength enhancement and performance prediction. For instance, Ma et al. [16] investigated the performance changes of steel slag aggregate after carbonation treatment with carbon dioxide, finding that carbonation significantly improved the compressive strength and volume stability of the aggregate, enabling it to meet Class II aggregate standards while simultaneously achieving CO_2_ sequestration. This provides a new pathway to synergistically enhance material performance and environmental benefits.

Regarding performance prediction, Wang et al. [17] employed an explainable machine learning approach to establish a predictive model for the compressive strength of sustainable cement–fly ash mortar. They found that the Gradient Boosting Regressor (GBR) offered the best prediction accuracy, with curing time being the most critical factor influencing strength. The Al_2_O_3_ content in fly ash was more influential than its dosage or the water-to-binder ratio. Zhang et al. [18] conducted research on recycled concrete incorporating superabsorbent polymer (SAP) and manufactured sand powder (MSP) under freeze–thaw conditions. They identified shifts in the dominant influencing factors before and after freeze–thaw cycles. They established a linear prediction model for strength loss, providing a basis for concrete design in harsh environments.

The regulatory mechanisms of admixtures on the cement hydration process and microstructure are key to the targeted optimization of material properties. Konan et al. [19] revealed the complex relationship between bubble dynamics and rheological behavior in cement paste through simulation, providing theoretical support for optimizing process parameters and reducing defects. Tao and Massoudi [20] studied the influence of nano-silica and fly ash on the pulsating flow behavior of cement suspensions, finding that they had opposing effects on flow velocity and particle distribution, with nano-silica dominating in composite systems.

Regarding microstructure enhancement, Shi et al. [21] investigated the stress–strain behavior and strength development of high-volume phosphogypsum-based cement materials. They indicated that increasing the slag content could significantly improve mechanical properties, and that sulfates in phosphogypsum promote ettringite formation, thereby enhancing system strength. Jiang et al. [22] studied the synergistic effect of MgO expansive agent and steel fibers, finding their combined use produced a “superposition effect,” markedly improving the crack resistance and mechanical properties of concrete, with uniform fiber distribution being a prerequisite for effective confinement and bridging.

Developing novel functional admixtures is a crucial pathway for advancing the high-performance and green development of cement-based materials. Dong et al. [23] synthesized hydrotalcite materials using steel slag as a raw material and verified their high-efficiency adsorption capacity for chloride and sulfate ions in salt-washing wastewater, demonstrating significant environmental benefits.

Furthermore, Ordonez et al. [24] investigated the regulatory effects of rice husk ash with varying reactivity on the performance of composite cements. The results showed that highly reactive rice husk ash, at appropriate dosages, could significantly improve cement performance and be combined with materials such as fly ash to prepare ternary systems with high replacement levels and high performance, providing technical support for the high-value utilization of agricultural waste.

The research compiled in this Special Issue showcases the latest progress in the field of cement admixtures from multiple dimensions: macroscopic performance enhancement, micro-mechanism interpretation, and development of novel additives. These works not only advance cement-based materials toward higher strength, durability, and functionality but also significantly enhance their environmental friendliness and resource-use efficiency. In the future, further revealing the intrinsic relationships among “composition–structure–performance–environment” through interdisciplinary approaches and intelligent methods will become an important development direction in this field. It is hoped that the articles in this issue will provide valuable references for researchers and engineering practice, jointly promoting the green innovation and sustainable development of cement technology.
